# Risk factors and prognosis for coronavirus disease 2019 among 131 hemodialysis patients during the Omicron variant epidemic

**DOI:** 10.1080/0886022X.2023.2228924

**Published:** 2023-07-28

**Authors:** Wen Wen, Shiming Cai, Yuehong Li, Xianglan Wu

**Affiliations:** Department of Nephrology, Beijing Tsinghua Changgung Hospital, School of Clinical Medicine, Tsinghua University, Beijing, China

**Keywords:** Omicron, COVID-19, hemodialysis, mortality

## Abstract

The present study evaluated the presentations and outcomes of coronavirus disease 2019 (COVID-19) among patients undergoing maintenance hemodialysis (MHD) and the impact of the Omicron BF.7 variant. Adult patients (age ≥ 18 years), who underwent MHD (dialysis vintage ≥ 3 months) at the Hemodialysis Center at Beijing Tsinghua Changgung Hospital between December 2022 and January 2023, were included based on predefined eligibility criteria. Clinical and laboratory characteristics were retrospectively collected. Among 131 patients who underwent MHD (10.7% vaccination rate), 106 (80.9%) tested positive for COVID-19. The prevalence of asymptomatic, mild, moderate, and severe COVID-19 was 8.5%, 58.5%, 17%, and 16%, respectively. Among the 97 patients with symptoms, 23 (23.7%) were hospitalized and six (5.7%) died. Fever was experienced by 74.2% of patients and respiratory symptoms were the most common (81.4%). Residual symptoms persisted in 20.9% of patients one month after the onset of COVID-19. COVID-19-positive hemodialysis patients were more likely to experience weight loss and exhibit reduced albumin levels compared to those without COVID-19 (*p* < .05). Compared with the asymptomatic group, patients with symptoms were younger, and exhibited higher interleukin-6 levels and lower post-infection phosphate levels (*p* < .05). Age, dialysis vintage, comorbidities, and inflammatory factors were positively associated with disease severity, while baseline albumin and hemoglobulin levels were associated with death (*p* < .05). In conclusion, COVID-19 was prevalent among patients undergoing MHD, even during the Omicron variant epidemic. Age, nutritional status, comorbidities, and inflammatory factors were associated with disease severity and prognosis.

## Introduction

Detection of the Omicron variant of the severe acute respiratory syndrome coronavirus-2 (SARS-CoV-2) was first reported on 24 November 2021, and quickly spread around the world. In December 2022, Beijing, China, witnessed a wave of SARS-CoV-2 infections dominated by the Omicron BF.7 variant. Weakened immune response, prolonged virus clearance, and low vaccination rate render patients undergoing hemodialysis particularly vulnerable [1–3]. A systematic review and meta-analysis investigating the mortality rate of coronavirus disease 2019 (COVID-19) among hemodialysis patients published in 2022 reported an infection rate of >10%, with a mortality rate of 20%. Biochemical abnormalities (e.g., inflammatory markers and electrolytes) and dyspnea have been generally reported to be associated with adverse outcomes [4]. However, as the virus evolves, the impact on patients undergoing hemodialysis may change accordingly.

To assess the impact of infection with the Omicron BF.7 variant on hemodialysis patients in the Chinese population, we analyzed the prevalence, clinical presentations, and outcomes of COVID-19 among patients undergoing maintenance hemodialysis (MHD) in our center.

## Patients and methods

### Patient selection

Adult patients (≥18 years of age), who underwent MHD (dialysis vintage ≥3 months) at the Hemodialysis Center in Beijing Tsinghua Changgung Hospital from December 2022 to January 2023, were included. Participants were excluded if related symptoms were not documented and/or could not be assessed. After excluding one patient who previously experienced severe complications and unconsciousness before COVID-19, 131 patients were included. Included patients were undergoing MHD 2–3 times per week using a dialysis machine (4008s, Fresenius Medical Care, Bad Homburg, Germany) and dialyzer (FX80, Fresenius, Bad Homburg, Germany), with a blood flow of 200–280 mL/min and a dialysate flow rate of 500 mL/min. The dialysate consisted of the following: sodium, 138–140 mmol/L; potassium, 2.0–3.0 mmol/L; calcium, 1.25–1.5 mmol/L; and magnesium, 0.5 mmol/L. All patients provided informed written consent to participate in the present study, which was approved by the Ethics Committee of Beijing Tsinghua Changgung Hospital, Tsinghua University.

### Data collection

Clinical and laboratory characteristics including demographic information (e.g., age, sex, dialysis vintage, and smoking), clinical comorbidities (diabetes mellitus (DM), coronary artery disease (CAD), hypertension, cerebrovascular disease (CVD), asthma, chronic obstructive pulmonary disease (COPD), malignancy, tuberculosis (TB)), laboratory results (e.g., hemoglobin, white blood cell count, albumin, creatinine, electrolytes, interleukin-6 (IL-6), and procalcitonin (PCT)) at baseline (within 3 months before COVID-19 diagnosis) and post-infection (within 1 month after infection), treatment (mainly regarding corticosteroids and specific antiviral therapies), and prognosis were retrospectively reviewed. The patients were interviewed face-to-face about their COVID-19 related symptoms at the time of infection and presently (at least 1 month after infection). Compared with baseline values, patients who experienced any reduction in their post-infection hemoglobin or albumin levels were considered to have decreased hemoglobulin or albumin. Clinical types and outcomes of patients diagnosed with COVID-19 were assessed.

The severity of COVID-19 was assessed according to the ‘Diagnosis and Treatment Protocol for Novel Coronavirus infection (Trial version 10)’. For patients with symptoms, mild COVID-19 could be diagnosed only if patients presented with upper respiratory symptoms (e.g., sore throat, cough, and fever). If imaging revealed typical COVID-19 pneumonia with any of respiratory rate >30 breaths/min, digital oxygen saturation ≤93%, partial pressure of oxygen (PaO_2_)/fraction of inspired oxygen (FiO_2_) ≤ 300 mmHg, or symptoms worsened progressively and lung imaging revealed that the lesion progressed significantly by >50% within 24–48 h, diagnosis of severe COVID-19 could be made. If imaging revealed typical COVID-19 pneumonia without the presentations above, patients were defined to have moderate COVID-19. Asymptomatic COVID-19 was diagnosed in those in whom symptoms were absent.

### Statistical analysis

Data analysis was performed using Stata release 16 (StataCorp LLC, College Station, TX). The prevalence of different presentations was tabulated and contrasted in plots. Characteristics of patients with different clinical types and outcomes were compared. Categorical data were analyzed using the Chi-squared test and quantitative data were analyzed using the *t*-test. Differences with *p* < .05 were considered to be statistically significant.

## Results

### Baseline characteristics and clinical presentations

Clinical information and laboratory data for 131 patients who underwent MHD in the authors’ center were collected. The mean (±SD) age of the cohort was 60.0 ± 15.3 years (range, 23–86 years). Mean dialysis vintage was 5.2 ± 4.3 years, and 62.6% of the patients were male. Only 14 (10.7%) patients had received CoronaVac or Sinopharm inactivated COVID-19 vaccine(s) (one dose (*n* = 1); two doses (*n* = 7); three doses (*n* = 3)). All final doses of vaccinations were received >1 month previously. Compared with those who did not receive vaccination, patients who were vaccinated were younger and less likely to have CAD (*p* < .05) (Supplementary Table 1).

**Figure 1.**
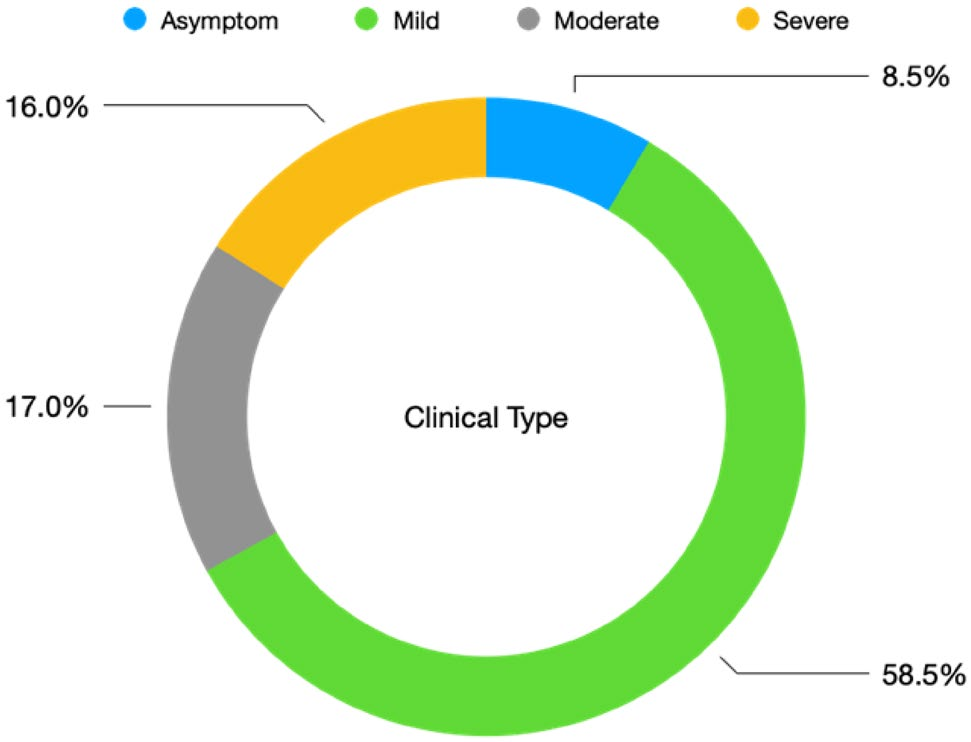
Distribution of clinical types among the 106 COVID-19 positive patients.

From December 1 to December 31, 106 (80.9%) patients tested positive for COVID-19. As shown in Figure 1, among the 106 COVID-19-positive patients, more than one-half (*n* = 62 (58.5%)) had a mild type of COVID-19, while moderate and severe COVID-19 pneumonia was recorded in 18 (17.0%) and 17 (16.0%) patients, respectively. Symptoms were absent in nine (8.5%) patients. Among the 97 patients with symptoms, 23 (23.7%) were hospitalized and 6 (5.7%) died.

**Figure 2.**
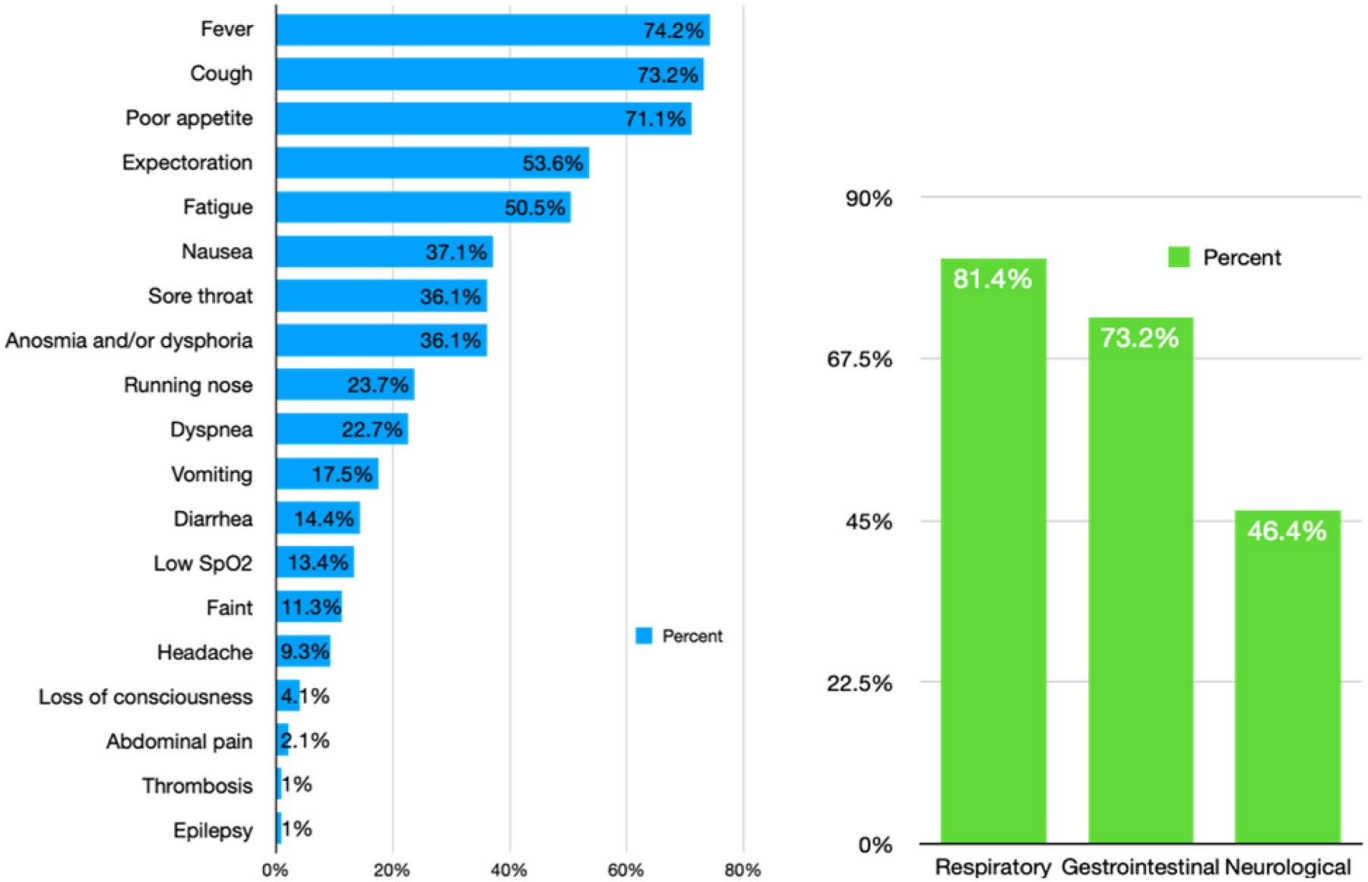
The distribution of symptoms in the 97 symptomatic COVID-19 patients.

Among the 97 COVID-19 patients with symptoms, fever was the most prevalent (74.2%), followed by cough (73.2%), poor appetite (71.1%), expectoration (53.6%), fatigue (50.5%), nausea (37.1%), sore throat (36.1%), anosmia or dysphoria (36.1%), runny nose (23.7%), dyspnea (22.7%), vomiting (17.5%), diarrhea (14.4%), low oxygen saturation (i.e., SpO_2_) (13.4%), lightheadedness (11.3%), headache (9.3%), loss of consciousness (4.1%), abdominal pain (2.1%), thrombosis (1%), and epilepsy (1%) (Figure 2(a)). When these symptoms were categorized into different systems, 81.4% of patients had respiratory, 73.2% had gastrointestinal, and 46.4% had neurological symptoms (Figure 2(b)).

### Residual symptoms

Among the 97 patients with symptoms, 6 died due to severe COVID-19 pneumonia. After at least one month after disease onset, 19 of the remaining 91 patients experienced persistent symptoms related to COVID-19, including fatigue (*n* = 12 (63.1%)), cough (*n* = 7 (36.8%)), poor appetite (*n* = 4 (21.1%)), and dyspnea (*n* = 2 (10.5%)).

**Figure 3.**
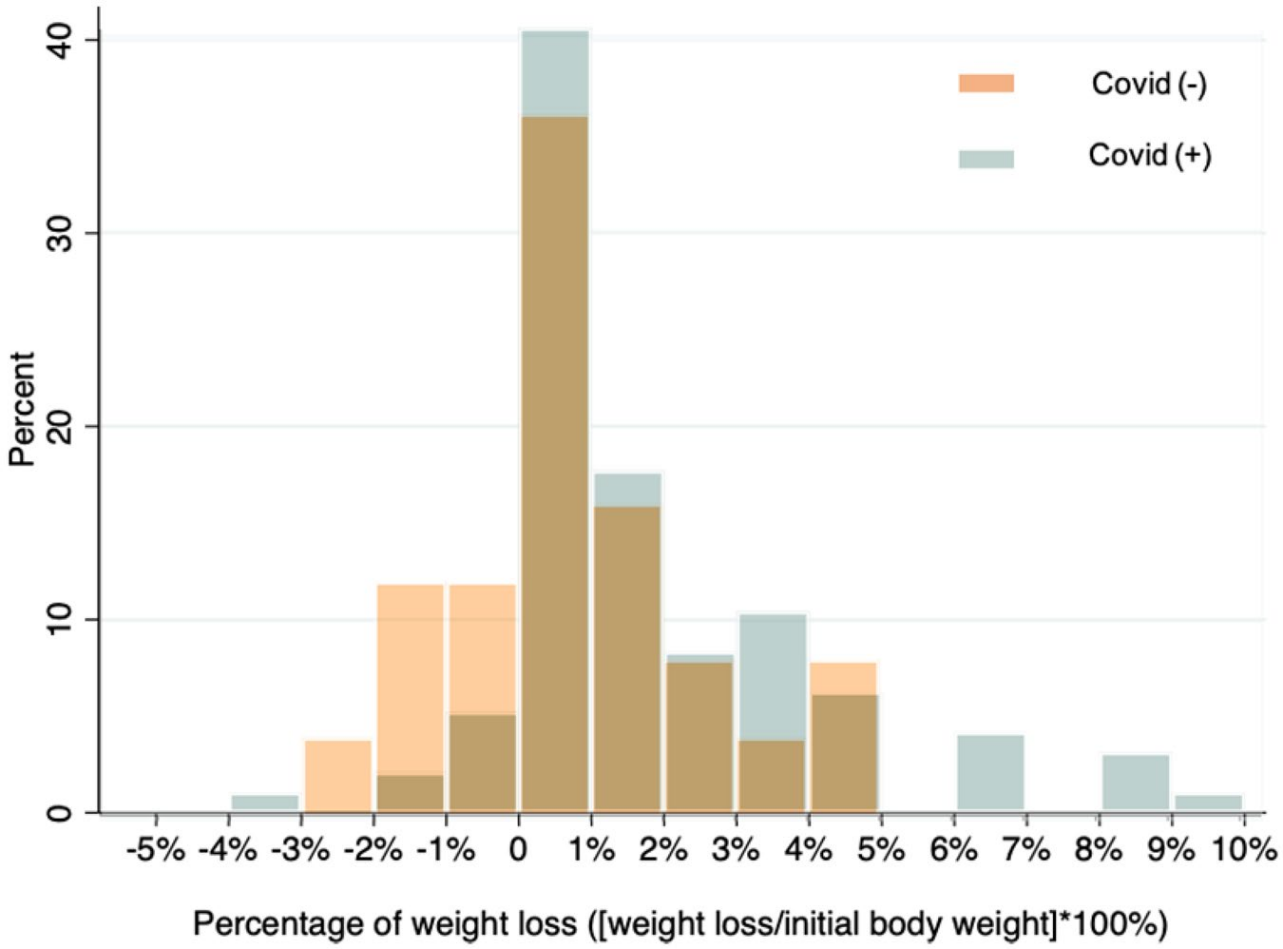
Distribution of weight loss in patients with and without COVID-19 infection.

Compared with COVID-19-negative patients, more COVID-19-positive patients experienced weight loss (69.8% vs. 48%; Chi-squared = 4.268, *p* = .039). The detailed distribution of dry weight reduction is shown in Figure 3. As presented in Figure 3, weight loss among COVID-19-positive patients exceeded that for COVID-19-negative patients in most of the categories except for 4–5%, with weight loss of 6–10% noted only in patients with COVID-19.

Changes in hemoglobulin and albumin levels were also compared between patients with and without COVID-19. Decreased albumin level was more common among patients with COVID-19 (27% vs. 4.3%; Chi-squared = 5.457, *p* = .019). However, no difference was noted in prevalence of hemoglobulin reduction between patients with and without COVID-19 (70% vs. 56.5%, respectively; Chi-squared = 1.548, *p* = .213).

### Comparisons between asymptomatic and symptomatic COVID-19 patients

Clinical characteristics were compared between COVID-19 patients with and without symptoms. Compared with the asymptomatic group, patients with COVID-19-related symptoms were younger, and exhibited higher IL-6 and lower post-infection phosphate levels (*p* < .05) (Table 1).

**Table 1.** Comparisons between asymptomatic and symptomatic patients with COVID-19.

	Asymptomatic group (*n* = 9)	Symptomatic group (*n* = 97)	*p* Value
Age, years	67.00 ± 7.762	58.44 ± 16.101	.013*
Dialysis vintage, years	5.72 ± 5.745	5.26 ± 4.243	.765
Male, *n* (%)	7 (77.8)	57 (58.8)	.265
Smoking, *n* (%)	4 (44.4)	45 (46.4)	.911
Hypertension, *n* (%)	8 (88.9)	88 (90.7)	.857
DM, *n* (%)	5 (55.6)	43 (44.3)	.518
CAD, *n* (%)	3 (33.3)	31 (32)	.933
CVD, *n* (%)	4 (44.4)	18 (18.6)	.067
COPD, *n* (%)	0 (0)	3 (3.1)	.592
Asthma, *n* (%)	0 (0)	1 (1.0)	.760
Malignancy, *n* (%)	0 (0)	12 (12.3)	.262
AID, *n* (%)	0 (0)	2 (2.1)	.867
TB, *n* (%)	1 (11.1)	4 (4.1)	.344
Inactivated COVID-19 vaccine, *n* (%)	0 (0)	11 (11.3)	.286
Baseline WBC, ×10^9^/L	6.92 ± 1.493	6.44 ± 1.901	.459
Baseline RBC, ×10^12^/L	3.83 ± 0.665	3.69 ± 0.466	.412
Baseline hemoglobulin, g/L	113.00 ± 14.841	112.17 ± 12.602	.853
Baseline platelets, ×10^9^/L	191.22 ± 72.586	187.02 ± 57.007	.837
Baseline total protein, g/L	70.13 ± 5.104	68.21 ± 4.791	.283
Baseline albumin, g/L	40.96 ± 3.995	41.43 ± 3.142	.689
Baseline serum creatinine, μmol/L	870.78 ± 267.735	1012.90 ± 273.653	.139
Baseline calcium, mmol/L	2.2 ± 0.267	2.23 ± 0.218	.861
Baseline phosphate, mmol/L	1.96 ± 0.526	1.86 ± 0.626	.653
Baseline potassium, mmol/L	5.00 ± 0.964	4.97 ± 0.591	.915
Baseline sodium, mmol/L	139.46 ± 3.062	139.22 ± 2.949	.816
Baseline CO_2_CP, mmol/L	22.01 ± 2.064	21.60 ± 2.721	.679
Post-infection WBC, ×10^9^/L	6.13 ± 2.428	6.28 ± 2.137	.837
Post-infection RBC, ×10^12^/L	3.75 ± 0.489	3.52 ± 0.447	.143
Post-infection hemoglobulin, g/L	109.00 ± 13.096	105.54 ± 13.97	.473
Post-infection platelets, ×10^9^/L	187.56 ± 59.750	182.68 ± 62.483	.823
Post-infection total protein, g/L	67.10 ± 1.838	63.61 ± 7.708	.533
Post-infection albumin, g/L	33.75 ± 0.070	34.98 ± 4.939	.730
Post-infection serum creatinine, μmol/L	951.11 ± 295.918	1000.47 ± 260.68	.593
Post-infection calcium, mmol/L	2.02 ± 0.205	2.14 ± 0.193	.096
Post-infection phosphate, mmol/L	2.37 ± 0.770	1.85 ± 0.718	.043*
Post-infection potassium, mmol/L	4.69 ± 0.911	4.77 ± 0.722	.758
Post-infection sodium, mmol/L	138.01 ± 7.12	138.48 ± 3.669	.737
Post-infection CO_2_CP, mmol/L	20.12 ± 3.704	22.14 ± 3.554	.108
Post-infection IL-6, pg/mL	14.23 ± 13.543	68.16 ± 174.384	.030*
Post-infection PCT, ng/mL	0.42 ± 0.085	2.39 ± 9.076	.631

DM: diabetes mellitus; CAD: coronary artery disease; CVD: cerebrovascular disease; COPD: chronic obstructive pulmonary disease; TB: tuberculosis; AID: autoimmune disease; IL-6: interleukin-6; PCT: procalcitonin; WBC: white blood cell; RBC: red blood cell; CO_2_CP: carbon dioxide binding capacity; baseline: data within three months before COVID-19 infection; post-infection: data within one month after COVID-19 infection.

*p < .05.

### Comparisons among different clinical types

Patient features were stratified and compared according to mild, moderate, and severe disease groups. Compared with the moderate COVID-19 group, patients with mild COVID-19 were younger, had a shorter dialysis vintage, a lower prevalence of DM, CAD, or TB, lower IL-6 and PCT levels, and higher post-infection albumin level (*p* < .05). A similar trend was observed in comparisons between patients with mild and severe COVID-19. Furthermore, hemoglobin and sodium levels were significantly lower in patients with severe COVID-19 compared with those in the mild group (*p* < .05), although no significant difference was noted between the moderate and severe groups. Regarding medications, corticosteroids and antiviral drugs were absent in patients with mild COVID-19 (Table 2). Only three of the 97 patients with symptoms had undergone specific antiviral therapies. Two patients in the moderate group received nirmatrelvir/ritonavir, whereas one in the severe group received azvudine, all of whom survived.

**Table 2.** The features of the patients with different severity of COVID-19.

	Mild group (*n* = 62)	Moderate group (*n* = 18)	Severe group (*n* = 17)	*p* Values		
				Mild vs. moderate	Mild vs. severe	Moderate vs. severe
Age (years)	52.13 ± 15.66	67.11 ± 9.628	72.29 ± 8.894	.000*	.000*	.108
Dialysis vintage (years)	4.40 ± 3.643	6.75 ± 4.697	6.82 ± 5.105	.028*	.030*	.965
Male, *n* (%)	35 (56.5)	10 (55.6)	12 (70.6)	.946	.293	.358
Smoking, *n* (%)	28 (45.2)	7 (38.9)	10 (58.8)	.637	.318	.238
Hypertension, *n* (%)	54 (87.1)	17 (94.4)	17 (100)	.385	.118	.324
DM, *n* (%)	21 (33.9)	11 (61.1)	11 (64.7)	.038*	.022*	.826
CAD, *n* (%)	13 (21)	9 (50)	9 (52.9)	.015*	.009*	.862
CVD, *n* (%)	8 (12.9)	4 (22.2)	6 (35.7)	.330	.032*	.392
COPD, *n* (%)	0 (0)	0 (0)	3 (17.6)	NA	.001*	.062
Asthma, *n* (%)	1 (1.6)	0 (0)	0 (0)	.588	.598	NA
Malignancy, *n* (%)	6 (9.7)	2 (11.1)	4 (23.5)	.858	.128	.330
TB (lung), *n* (%)	0 (0)	2 (11.1)	2 (11.8)	.008*	.006*	.952
Vaccine, *n* (%)	11 (17.7)	0 (0)	0 (0)	.054	.061	NA
Corticosteroid, *n* (%)	0 (0)	11 (61.1)	9 (52.9)	.000*	.000*	.625
Antiviral therapy^a^, *n* (%)	0 (0)	2 (11.0)	1 (5.9)	.008*	.055	.581
Baseline WBC, ×10^9^/L	6.47 ± 1.976	6.80 ± 1.585	5.95 ± 1.947	.521	.343	.167
Baseline RBC, ×10^12^/L	3.71 ± 0.472	3.77 ± 0.284	3.53 ± 0.577	.606	.193	.124
Baseline hemoglobulin, g/L	111.95 ± 13.732	114.83 ± 6.724	110.12 ± 13.374	.394	.628	.193
Baseline platelets, ×10^9^/L	191.20 ± 61.738	184.83 ± 61.555	174.82 ± 28.612	.702	.294	.204
Baseline total protein, g/L	68.41 ± 4.853	68.53 ± 4.437	67.20 ± 5.095	.927	.386	.427
Baseline albumin, g/L	41.80 ± 2.736	41.28 ± 3.440	40.313 ± 3.995	.517	.088	.460
Baseline serum creatinine, μmol/L	1042.17 ± 288.694	1013.50 ± 198.968	910.71 ± 277.189	.695	.099	.214
Baseline calcium, mmol/L	2.26 ± 0.222	2.21 ± 0.123	2.17 ± 0.272	.338	.190	.652
Baseline phosphate, mmol/L	1.86 ± 0.649	1.91 ± 0.702	1.82 ± 0.469	.775	.792	.640
Baseline potassium, mmol/L	4.98 ± 0.613	5.05 ± 0.662	4.87 ± 0.433	.681	.500	.359
Baseline sodium, mmol/L	139.44 ± 2.708	138.55 ± 3.462	139.18 ± 3.248	.260	.747	.581
Baseline CO_2_CP, mmol/L	21.81 ± 2.448	21.21 ± 2.858	21.30 ± 3.493	.385	.496	.935
Post-infection WBC, ×10^9^/L	6.15 ± 1.966	6.53 ± 2.767	6.47 ± 2.028	.523	.574	.942
Post-infection RBC, ×10^12^/L	3.60 ± 0.453	3.41 ± 0.404	3.32 ± 0.405	.115	.025*	.488
Post-infection hemoglobulin, g/L	108.25 ± 13.606	101.67 ± 12.848	100.25 ± 13.723	.074	.042*	.758
Post-infection platelets, ×10^9^/L	186.91 ± 62.770	188.56 ± 60.945	161.00 ± 62.408	.923	.148	.202
Post-infection total protein, g/L	67.12 ± 5.301	62.44 ± 5.830	60.46 ± 9.760	.074	.202	.995
Post-infection albumin, g/L	40.13 ± 3.538	34.73 ± 2.985	31.88 ± 4.450	.001*	.000*	.072
Post-infection serum creatinine, μmol/L	1053.63 ± 248.609	939.72 ± 232.091	879.44 ± 291.790	.090	.020*	.508
Post-infection calcium, mmol/L	2.17 ± 0.190	2.11 ± 0.128	2.06 ± 0.244	.220	.071	.491
Post-infection phosphate, mmol/L	1.91 ± 0.708	1.71 ± 0.855	1.79 ± 0.569	.323	.550	.772
Post-infection potassium, mmol/L	4.85 ± 0.699	4.66 ± 0.705	4.58 ± 0.810	.309	.182	.755
Post-infection sodium, mmol/L	139.17 ± 3.094	137.85 ± 4.712	136.78 ± 3.804	.277	.012*	.476
Post-infection CO_2_CP, mmol/L	22.49 ± 2.937	22.55 ± 4.425	22.44 ± 4.205	.945	.029*	.165
Post-infection IL-6, pg/mL	7.52 ± 6.435	54.76 ± 70.197	217.83 ± 317.140	.026*	.034*	.093
Post-infection PCT, ng/mL	0.73 ± 0.733	1.3 ± 1.009	7.58 ± 18.786	.032*	.196	.207

DM: diabetes mellitus; CAD: coronary artery disease; CVD: cerebrovascular disease; COPD: chronic obstructive pulmonary disease; TB: tuberculosis; AID: autoimmune disease; IL-6: interleukin-6; PCT: procalcitonin; WBC: white blood cell; RBC: red blood cell; CO_2_CP: carbon dioxide binding capacity; baseline: data within three months before COVID-19 infection; post-infection: data within one month after COVID-19 infection.

*p < .05.

^a^ The two patients in the moderate group received nirmatrelvir/ritonavir while the one in the severe group received azvudine.

Regarding COVID-19-related symptoms, the prevalence of dyspnea significantly increased with disease severity (*p* < .05). Loss of consciousness was only observed in patients with severe COVID-19. The prevalence of an upper respiratory symptom (runny nose) decreased from mild to severe COVID-19, and a significantly lower prevalence of runny nose was noted in patients with severe COVID-19 compared to those with mild disease (*p* = .048) (Table 3).

**Table 3.** Symptoms among patients with different severity of COVID-19.

	Mild (*n* = 62)	Moderate (*n* = 18)	Severe (*n* = 17)	*p* Value		
				Mild vs. moderate	Mild vs. severe	Moderate vs. severe
Fever, *n* (%)	44 (71)	15 (83.3)	13 (76.5)	.294	.654	.612
Sore throat, *n* (%)	21 (33.9)	9 (50)	5 (29.4)	.213	.729	.214
Cough, *n* (%)	45 (72.6)	15 (83.3)	11 (64.7)	.354	.527	.208
Expectoration, *n* (%)	31 (50)	12 (66.7)	9 (52.9)	.212	.830	.407
Running nose, *n* (%)	18 (29)	4 (22.2)	1 (5.9)	.569	.048*	.167
Dyspnea, *n* (%)	2 (3.2)	6 (33.3)	14 (82.4)	.000*	.000*	.003*
Hypoxemia, *n* (%)	0 (0)	1 (5.6)	12 (70.6)	.062	.000*	.000*
Anorexia, *n* (%)	41 (66.1)	15 (83.3)	13 (76.5)	.161	.417	.612
Nausea, *n* (%)	21 (33.9)	8 (44.4)	7 (41.2)	.411	.557	.845
Vomiting, *n* (%)	9 (14.5)	4 (22.2)	4 (23.5)	.435	.375	.927
Diarrhea, *n* (%)	9 (14.5)	4 (22.2)	1 (5.9)	.435	.343	.167
Abdominal pain, *n* (%)	1 (1.6)	1 (5.6)	0 (0)	.346	.598	.324
Thrombosis, *n* (%)	0 (0)	1 (5.6)	0 (0)	.062	NA	.324
Faint, *n* (%)	8 (12.9)	1 (5.6)	2 (11.8)	.385	.900	.512
Headache, *n* (%)	4 (6.5)	4 (22.2)	1 (5.9)	.050	.932	.167
Epilepsy, *n* (%)	0 (0)	0 (0)	1 (5.9)	NA	.055	.296
Loss of consciousness, *n* (%)	0 (0)	0 (0)	4 (23.5)	NA	.000*	.029*
Anosmia, *n* (%)	21 (33.9)	8 (44.4)	6 (35.3)	.411	.913	.581

*p < .05.

### Characteristics associated with survival

Clinical features and laboratory data from patients who survived and those who did not are summarized in Table 4. Patients who died were older, more likely to have DM and/or COPD, and exhibited higher IL-6 or PCT levels (*p* < .05). Nevertheless, non-survivors exhibited lower baseline red blood cell counts and serum albumin levels (*p* < .05).

**Table 4.** Comparisons between survivors and non-survivors after COVID-19 infection.

	Survivor (*n* = 91)	Non-survivor (*n* = 6)	*p* Value
Age (years)	57.27 ± 15.801	76.17 ± 9.020	.005*
Dialysis vintage (years)	5.09 ± 4.043	7.83 ± 6.554	.127
Male, *n* (%)	54 (59.3)	3 (50)	.653
Smoking, *n* (%)	43 (47.3)	2 (33.3)	.508
Hypertension, *n* (%)	82 (90.1)	6 (100)	.419
DM, *n* (%)	38 (41.8)	5 (83.3)	.047*
CAD, *n* (%)	28 (30.8)	3 (50)	.328
CVD, *n* (%)	16 (17.6)	2 (33.3)	.336
COPD, *n* (%)	1 (1.1)	2 (33.3)	.000*
Asthma, *n* (%)	1 (1.1)	0 (0)	.796
Malignancy, *n* (%)	11 (12.1)	1 (16.7)	.741
AID, *n* (%)	2 (2.2)	0 (0)	.714
TB, *n* (%)	4 (4.4)	0 (0)	.600
Vaccine, *n* (%)	11 (12.1)	0 (0)	.366
Baseline WBC, ×10^9^/L	6.42 ± 1.882	6.73 ± 2.348	.695
Baseline RBC, ×10^12^/L	3.72 ± 0.439	3.25 ± 0.658	.014*
Baseline hemoglobulin, g/L	112.52 ± 12.308	107.00 ± 16.840	.301
Baseline platelets, ×10^9^/L	187.80 ± 58.380	175.67 ± 31.284	.617
Baseline total protein, g/L	68.34 ± 4.599	66.53 ± 7.313	.375
Baseline albumin, g/L	41.64 ± 2.800	38.56 ± 5.882	.020*
Baseline serum creatinine, μmol/L	1026.99 ± 268.592	806.33 ± 287.955	.056
Baseline calcium, mmol/L	2.24 ± 0.207	2.09 ± 0.333	.103
Baseline phosphate, mmol/L	1.86 ± 0.645	1.92 ± 0.216	.816
Baseline potassium, mmol/L	4.99 ± 0.594	4.67 ± 0.497	.193
Baseline sodium, mmol/L	139.19 ± 3.023	139.63 ± 1.560	.728
Baseline CO_2_CP, mmol/L	21.58 ± 2.715	21.95 ± 3.040	.749
Post-infection IL-6, pg/mL	36.00 ± 86.204	486.23 ± 424.513	.000*
Post-infection PCT, ng/mL	1.10 ± 1.084	17.35 ± 30.925	.000*

DM: diabetes mellitus; CAD: coronary artery disease; CVD: cerebrovascular disease; COPD: chronic obstructive pulmonary disease; TB: tuberculosis; AID: autoimmune disease; IL-6: interleukin-6; PCT: procalcitonin; WBC: white blood cell; RBC: red blood cell; CO_2_CP: carbon dioxide binding capacity; baseline: data within three months before COVID-19 infection; post-infection: data within one month after COVID-19 infection.

*p < .05.

It was noteworthy that none of the patients with severe COVID-19 received a COVID vaccine. As shown in Supplementary Table 2, among the 17 patients with severe COVID-19, the six patients who died exhibited significantly higher IL-6 levels compared with those who survived (486.2 ± 424.5 pg/mL vs. 98.6 ± 176.8 pg/mL, respectively; *p* = .035).

## Discussion

This is the first study to focus on the impact of COVID-19 on hemodialysis patients in China following the outbreak of Omicron BF.7 infection in Beijing. In this single-center study, we found that the prevalence of COVID-19 was high (80.9%) among hemodialysis patients, more than one-half (58%) of whom had mild disease. Pneumonia occurred in 33% as seen in those with moderate or severe disease. The mortality rate was 5.7%, which is relatively low compared to previous reports [4]. Respiratory symptoms were still the most common presentation among patients with COVID-19. Disease severity was associated with age, dialysis vintage, comorbidities, and IL-6 levels. Weight loss, decreased albumin level, and residual symptoms were observed in COVID-19-positive patients.

Characteristics of the virus that causes COVID-19 have been shifting over time. Among hematology patients, mortality and the rate of post-COVID-19 syndrome have declined with time across SARS-CoV-2 variants [5]. According to a large retrospective study conducted in Spain, infection by the Omicron variant was associated with higher reinfection rates and lower disease severity (28-day hospital mortality) than previous variants [6]. In 2020, Neumann-Podczaska et al. reported a significant proportion of patients (25.7%) developing acute renal injury and high need for oxygen supplementation (71.4%) after COVID-19 [7]. Compared with previous SARS-CoV-2 variants, Omicron mainly targets the upper respiratory tract among patients across different ages regardless of vaccination status [6]. Banshodani et al. summarized the characteristics of COVID-19 among hemodialysis patients in Japan and similar mortality rate (6.8%) as that in our study was reported [8]. However, higher mortality rate has been observed in hospitalized COVID-19 patients on MHD in Shanghai (11.8%), probably due to the higher proportion of patients suffering from COVID-19 pneumonia (43%) in hospitalized patients [9].

Nevertheless, elderly patients remain susceptible to infection in high-risk settings (e.g., hospitals, geracomium) [10]. When infected, elderly patients are more likely to progress to severe COVID-19 pneumonia and acquire infections from the other microbes or systems [11]. In our study, severe COVID-19 was significantly associated with age, reflecting the unchanged vulnerability of the elderly population of hemodialysis patients in the Omicron era. Post-infection symptoms were related to the extent of the immune response. Elderly patients may have a weak immune system and yield to minor symptoms, which must be noticed and addressed in time [12]. In our study, age and upper respiratory symptoms were both associated with severe COVID-19 pneumonia but in different directions, indicating that age-associated immune response plays an important role in fighting the virus that causes COVID-19. Furthermore, underlying comorbidities (e.g., DM, CAD, and TB) were also risk factors for the development of COVID-19 pneumonia among hemodialysis patients. Additionally, younger patients experienced fewer complications and exhibited higher vaccination rates, which are both associated with milder presentations and better prognoses [13,14].

Poor nutritional status is associated with infection, anemia, and high hospitalization and mortality rates [15]. Previous studies have reported that patients with poor nutritional status had a high risk for progressing to severe type pneumonia and poor prognosis [16]. In our analysis, low albumin and a reduction in albumin level were both found in patients with COVID-19, especially those with severe pneumonia. Lower post-infection phosphate and prolonged poor appetite were found in COVID-19 patients with symptoms, indicating that nutritional status could be worsened by the COVID-19 disease, which could further impact immune response and exacerbate the situation.

Several studies have reported the high prevalence of hyponatremia after COVID-19 [17]. Possible reasons include decreased appetite, decreased sodium salt intake, electrolyte loss from vomiting and diarrhea, or increased water intake for fever. Our study also observed that the post-infection sodium level decreased, and there was a significant difference between the severe and mild disease groups. Among hemodialysis patients, hyponatremia is associated with a higher mortality rate [17]. The level and change(s) in inflammatory factors have been demonstrated to reflect the prognosis of COVID-19 [4]. IL-6 and PCT have been associated with COVID-19-related in-hospital death, and were predictors of all-cause mortality in dialysis patients [18,19]. In our study, non-survivors exhibited significantly higher PCT and IL-6 levels than survivors. It is proposed that another inflammatory factor–serum chemokine CC-motif ligand 17, is a good predictive marker of severe COVID-19 in hemodialysis patients [8,20]. Monitoring inflammation markers to better recognize patients at high risk for death, especially those with minor presentations, is necessary for treating COVID-19.

Vaccination has been demonstrated to be safe and effective in both hemodialysis patients and elderly individuals [21]. However, a low vaccination rate was observed in our hemodialysis center, which is coincident with the high rate of hesitancy toward primary COVID-19 vaccination worldwide. Egyptian researchers surveyed reasons for denying boosters for COVID-19 in hemodialysis patients. They found that more than one-half of the patients did not want to receive boosters, and booster hesitancy was associated with female sex, younger age, single marital status, Alexandria and urban residency, and the use of a tunneled dialysis catheter [22].

The availability of antiviral therapies for the patients undergoing hemodialysis is extremely limited. Most clinical trials investigating COVID-19 antiviral drugs have not focused on patients with end-stage renal disease [23]. Nirmatrelvir/ritonavir has not been widely used in patients undergoing hemodialysis in China because it is not recommended for patients with an estimated glomerular filtration rate (eGFR) < 30 mL/min*1.73 m^2^ [24]. Moreover, evidence is lacking for Azvudine among patients with an eGFR < 70 mL/min*1.73 m^2^ [25]. In our study, three patients received COVID-19 antiviral drugs and no adverse effect(s) was observed. In a study investigating the pharmacokinetics of nirmatrelvir/ritonavir in four patients undergoing MHD, no overdose or accumulation was noted [26]. To further test the efficacy and safety of antiviral COVID-19 drugs in this population, large randomized controlled trials among hemodialysis patients are warranted.

There were several limitations to our study, the first of which was its single-center design. Furthermore, individuals were asked to retrospectively report their symptoms, which may have introduced recall bias. More importantly, the sample size was limited, and uneven comparisons were made between different groups of patients. Due to the limited sample size, multiple comparisons were made using univariate regression without adjusting for confounders. In the future, multicenter studies with larger sample sizes are needed to further guide treatment and prevention.

## Conclusions

Despite the lower mortality rate compared with previous variants, one-half of hemodialysis patients in this study acquired viral pneumonia during the Omicron BF.7 epidemic. Age, nutritional status, comorbidities, and inflammatory factors were associated with the severity and prognosis of COVID-19 among patients undergoing MHD.

## Supplementary Material

Supplemental Material

## Data Availability

The data used or analyzed in the study are available from the corresponding author on reasonable request.
