# Supplementary material for: Risk factors and prognosis for coronavirus disease 2019 among 131 hemodialysis patients during the Omicron variant epidemic
Source: Ren Fail. 2023 Jul 28;45(1):2228924. doi: 10.1080/0886022X.2023.2228924 (PMC10388811; doi:10.1080/0886022X.2023.2228924)
Supplement: Supplemental Material [file IRNF_A_2228924_SM5884.pdf]

Supplementary Table 1: Comparisons between COVID-19 patients who were vaccinated and not vaccinated.

Factor	Level	Not vaccinated	Vaccinated	p-value
N		95	11	
Age, mean (SD)		61.2 (13.9)	41.3 (19.6)	<0.001
Gender (n, %)	Female	39 (41%)	3 (27%)	0.38
	Male	56 (59%)	8 (73%)	
Smoke (n, %)		43 (45%)	6 (55%)	0.56
Hypertention (n, %)		87 (92%)	9 (82%)	0.29
Diabetes (n, %)		46 (48%)	2 (18%)	0.056
CAD (n, %)		34 (36%)	0 (0%)	0.016
CVD (n, %)		21 (22%)	0 (0%)	0.082
COPD (n, %)		3 (3%)	0 (0%)	0.55
Asthma (n, %)		1 (1%)	0 (0%)	0.73
Malignancy (n, %)		11 (12%)	1 (9%)	0.81
AID (n, %)		2 (2%)	0 (0%)	0.63
Tuberculosis (n, %)		5 (5%)	0 (0%)	0.44

Information Classification: General

Supplementary Table 2: Comparisons between patients with severe COVID-19 who were survived and not survived.

Factor	Survived	Not survived	p-value
N	11	6	
Age, mean (SD)	70.2 (8.5)	76.2 (9.0)	0.19
Gender (n, %)	9 (82%)	3 (50%)	0.17
Smoke, (n, %)	8 (73%)	2 (33%)	0.11
Hypertention, (n, %)	11 (100%)	6 (100%)	
Diabetes, (n, %)	6 (55%)	5 (83%)	0.24
CAD	6 (55%)	3 (50%)	0.86
CVD	3 (27%)	2 (33%)	0.79
COPD	1 (9%)	2 (33%)	0.21
Corticosteroids	6 (55%)	3 (50%)	0.86
Antibiotics	11 (100%)	5 (83%)	0.16
COVID-19 antiviral drugs	1 (9%)	0 (0%)	0.45
IL-6, mean (SD)	98.6 (176.8)	486.2 (424.5)	0.035
PCT, mean (SD)	2.1 (1.6)	17.3 (30.9)	0.15
WBCpre, mean (SD)	5.5 (1.7)	6.7 (2.3)	0.23
RBCpre, mean (SD)	3.7 (0.5)	3.2 (0.7)	0.13
HGBpre, mean (SD)	111.8 (11.6)	107.0 (16.8)	0.50
PLTpre, mean (SD)	174.4 (28.6)	175.7 (31.3)	0.93
ALBpre, mean (SD)	41.4 (2.0)	38.6 (5.9)	0.18
Scrpre, mean (SD)	967.6 (267.2)	806.3 (288.0)	0.26
Uapre, mean (SD)	431.9 (101.6)	406.7 (62.1)	0.59
Capre, mean (SD)	2.2 (0.2)	2.1 (0.3)	0.38
Ppre, mean (SD)	1.8 (0.6)	1.9 (0.2)	0.52
Kpre, mean (SD)	5.0 (0.4)	4.7 (0.5)	0.16
Napre, mean (SD)	138.9 (3.9)	139.6 (1.6)	0.69
C02pre, mean (SD)	20.9 (3.8)	21.9 (3.0)	0.59

Information Classification: General
